# Inotilone from *Inonotus linteus* suppresses lung cancer metastasis *in vitro* and *in vivo* through ROS-mediated PI3K/AKT/MAPK signaling pathways

**DOI:** 10.1038/s41598-019-38959-z

**Published:** 2019-02-20

**Authors:** Wei Chao, Jeng-Shyan Deng, Pei-Ying Li, Yueh-Hsiung Kuo, Guan-Jhong Huang

**Affiliations:** 1 School of Chinese Pharmaceutical Sciences and Chinese Medicine Resources, College of Chinese Medicine, China Medical University, Taichung, 404 Taiwan; 2 Graduate Institute of Medical Sciences, College of Medicine, Taipei Medical University, Taipei, Taiwan; 3 Department of Health and Nutrition Biotechnology, College of Medical and Health Science, Asia University, Taichung, 413 Taiwan; 4 School of Pharmacy, College of Pharmacy, China Medical University, Taichung, 404 Taiwan

## Abstract

Metastasis is one of the main causes of mortality in cancer patients. Inotilone, a major component of *Inonotus linteus*, is a traditional Chinese medical herb. In this study, MTT results showed that inotilone had no obvious cytotoxicity. Animal model results revealed that inotilone suppressed cancer metastatic efficacy. Serum results showed that inotilone reduced the activity of matrix metalloproteinase (MMP)-2 and -9 and tumor necrosis factor alpha (TNF-α) activity as well as NO content. Additionally, inotilone affected MMP-9 and tissue inhibitor of metalloproteinase (TIMP)-2 protein expression and improved the activity of the antioxidant enzymes in the lung tissues of LLC-bearing mice. In addition, cell experimental results showed that inotilone reduced the activity of MMP-2/-9 and inhibited the ability for cellular migration and invasion. Inotilone decreased interleukin (IL)-8 expression in A549 cells. Western blot results revealed that inotilone affected the protein expression of MMPs, nitric oxide synthase (iNOS), cyclooxygenase (COX)-2, anti-oxidant enzymes, mitogen activated protein kinase (MAPK), focal adhesion kinase (FAK), phosphoinositide-3 kinase (PI3K)-AKT, and nuclear factor (NF)κB. Therefore, we propose that inotilone is a potential therapeutic candidate against metastatic lung cancer cells.

## Introduction

Lung cancer is one of the most common and fatal forms of malignancy in Taiwan. Because of its propensity for metastasis, lung cancer patients have a poor prognosis and high rates of treatment failure and death^1^. Therefore, how to inhibit cancer metastasis is becoming an important theme in clinical therapy. Cancer cell metastasis involves a complex process, including invasion, intravasation, colonization and angiogenesis^2^. Metastatic cancer cells have a critical characteristic, the ability to degrade the basement membranes and extracellular matrix (ECM). This degradative process is mediated by matrix metalloproteinases (MMPs)^3^, which are a family of zinc-dependent neutral endopeptidases^4^. Among all MMPs, MMP-2 and -9 degrade most constituents of the ECM directly and are involved in cancer metastasis^5^. The tumor microenvironment such as tumor necrosis factor alpha (TNF-α) secretion and stimuli by macrophages, can also increase the expression of MMPs^6^. Tissue inhibitors of metalloproteinases (TIMPs) are specific inhibitors of MMPs, and an imbalance between MMPs and TIMPs may contribute to the degradation or deposition of ECM^7^.

The MAPK pathway is considered to be one of the major mechanisms of relaying extracellular signals triggered by growth factors and cytokines to induce specific responses and genes. These signaling cascades play important roles in the regulation of cell growth, differentiation, apoptosis, and metastasis^8^. There is evidence that MMP expression is regulated by transcription factors through upstream pathways, including the MAPK and phosphoinositide-3 kinase (PI3K)-AKT pathways^9,10^.

The correlation between inflammation and cancer is not novel. In 1863, Virchow hypothesized that the origin of cancer was at sites of chronic inflammation^11^. Traditionally, reactive oxygen species (ROS), mainly consisting of superoxide anion radicals, singlet oxygen, hydrogen peroxide and the highly reactive hydroxyl radicals, were simply viewed as a group of molecules harmful to cells, tissues, and organisms. However, it is now clear that it is not just the proliferation of cells that causes cancer but also that sustained cell proliferation in an environment enriched with inflammatory cells, growth factors, activated stroma, and DNA damage-promoting agents potentiates and/or promotes neoplastic risk^11^. Therefore, some evidence indicates that metastasis can be inhibited by targeting anti-oxidant enzymes^12^.

*Inonotus linteus* (IL) is a species of mushroom belonging to the Hymenochaetaceae family that is popular in Asian countries. IL is usually used as food and medicine because it contains many bioactive compounds that possess many pharmacological actions, such as anti-tumor, anti-angiogenic and immunomodulatory properties^13^. Inotilone is an unusual 5-methyl-3(2 H)-furanone derivative that has been previously shown to be a potent inflammatory inhibitor that inhibits LPS-induced NO and PGE_2_ production by modulating nitric oxide synthase (iNOS) expression and cyclooxygenase (COX)-2 enzyme activity, respectively^14^. However, the anti-metastatic efficacy of inotilone is ambiguous. In this study, we used an animal model and cell experiments to investigate the efficacy of inotilone in anti-metastasis and the possible mechanism.

## Results

### Inotilone from IL and its structural characterization

**Figure 1:**
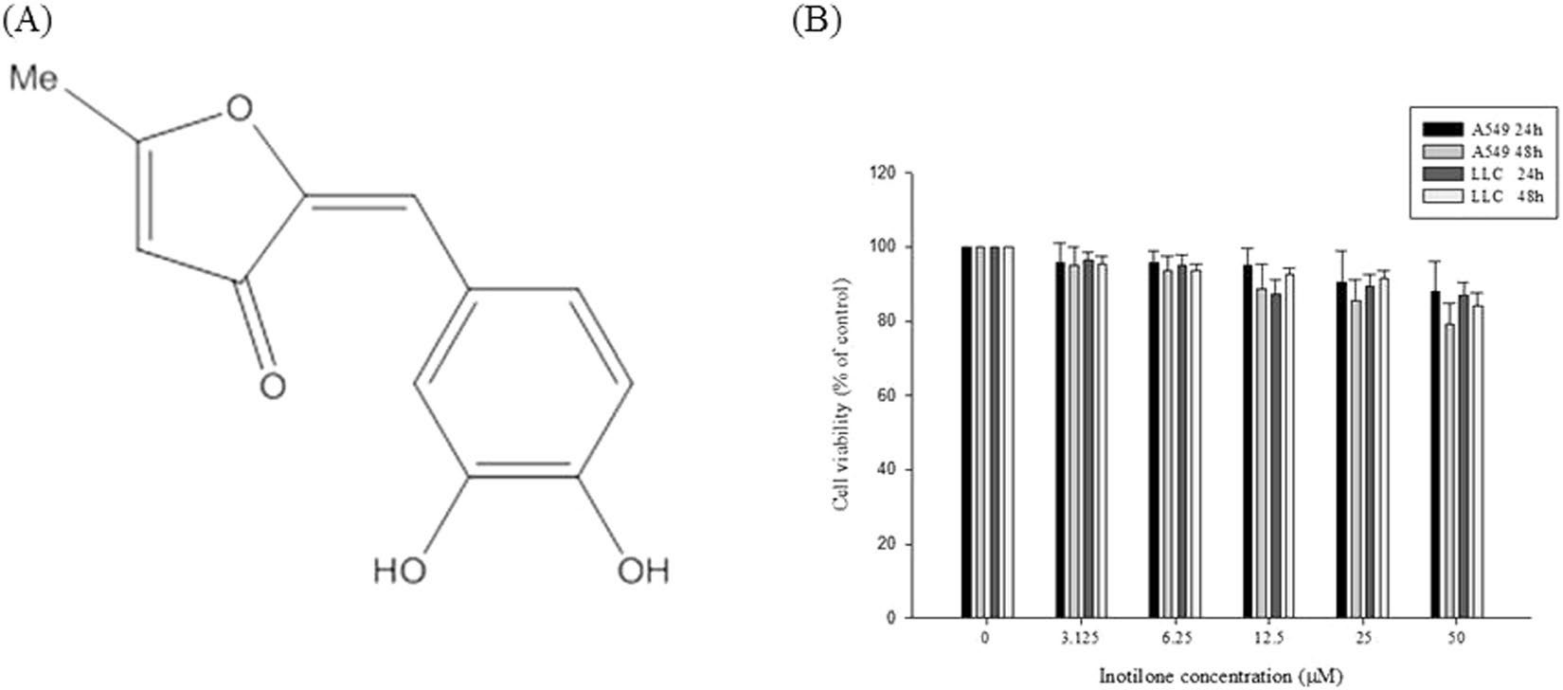
Effects of inotilone on the cell viability of A549 and LLC cells. (**A**) The chemical structure of inotilone. (**B**) A549 and LLC cells were seeded into 96-well plates and treated with different concentrations (0, 3.125, 6.25, 12.5, 25, and 50 μM) of inotilone for 24 and 48 h, and then, cell viability was determined by MTT assay. Data represent the mean ± SD from three independent experiments.

Inotilone was isolated from the ethyl acetate (EA) fraction (Fig. 1A). The chemical structure was elucidated by Nuclear Magnetic Resonance (NMR) spectroscopy. The mass spectrometry studies identified it as inotilone The spectral data of the isolated substance were as follows: ^1^H NMR (DMSO, 400 MHz) δ 2.55 (s, 3H, CH_3_), 5.80 (s, 1H, CH), 6.49 (s, 1H, CH), 6.80 (d, 1H, *J* = 8.4 Hz, ArH), 7.16 (dd, 1H, *J* = 8.4, 2.0 Hz, ArH), 7.34 (d, 1H, *J* = 2.0 Hz, ArH); ^13^C NMR (100 MHz, DMSO) δ 15.9, 105.7, 112.3, 116.2, 118.2, 123.1, 125.0, 144.6, 145.7, 148.4, 180.9, 187.0. These data match the reported literature values^15^.

### Cytotoxicity of inotilone in A549 and LLC cells

The cytotoxicity of inotilone was determined by MTT assay. The results revealed that inotilone had no obviously cytotoxicity in various cell lines (Fig. S1). Furthermore, we demonstrated that A549 and LLC cells treated with various concentrations of inotilone had no change in viability (Fig. 1B) compared to untreated controls.

### Inotilone inhibits lung cancer metastasis *in vivo*

**Figure 2:**
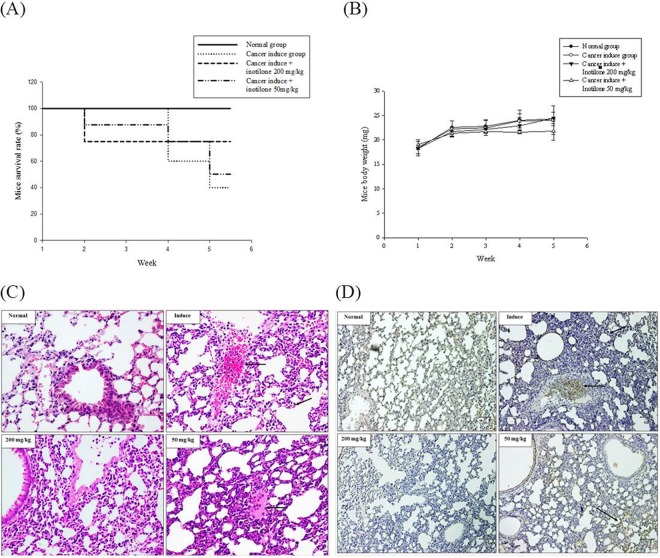
Inotilone suppressed cell metastasis *in vivo*. (**A**) LLC cells were injected subcutaneously into the left flank of C57BL/6 mice. The survival rates of each group for 5 weeks. (**B**) The body weights of each group were measured every week. After administration of inotilone (50 mg/kg and 200 mg/kg) for twenty one days, the mice were sacrificed, and the lung tissues were collected. (**C**) Effects of inotilone on metastasized tumor in lung tissues of C57BL/6 mice. H&E staining revealed the neutrophil infiltration and bleeding sections (arrow). (**D**) The proliferating cellular nuclear antigen (PCNA) expression in the lung tissue of LLC-bearing mice (arrow). The magnification was 400X.

LLC cells were injected subcutaneously into the left flank of C57BL/6 mice. To inhibit cancer cell metastasis, we intraperitoneally injected inotilone for 21 days. At the end of the experiments, a high dose of inotilone improved the survival rate of the LLC-bearing mice (Fig. 2A). The body weights of the C57BL/6 mice did not change in any of the four groups (Fig. 2B). The anti-metastatic effect of inotilone on LLC-induced metastasis could be confirmed by hematoxylin and eosin (H&E) staining and immunohistochemistry (IHC) for PCNA. The lung tissues in the tumor control group displayed an extensive coalescing area and neutrophilic infiltration in the interstitial area. Moreover, IHC staining revealed a proliferation area in the PCNA positive cells. In contrast, in the inotilone treatment groups, metastatic symptoms and proliferation cells were both attenuated, both in the extensive and neutrophilic infiltrated areas (Fig. 2C,D).

### Effects of inotilone on the activities of MMP-2 and MMP-9 and the expression of NO and TNF-α in the serum of LLC-bearing mice

**Figure 3:**
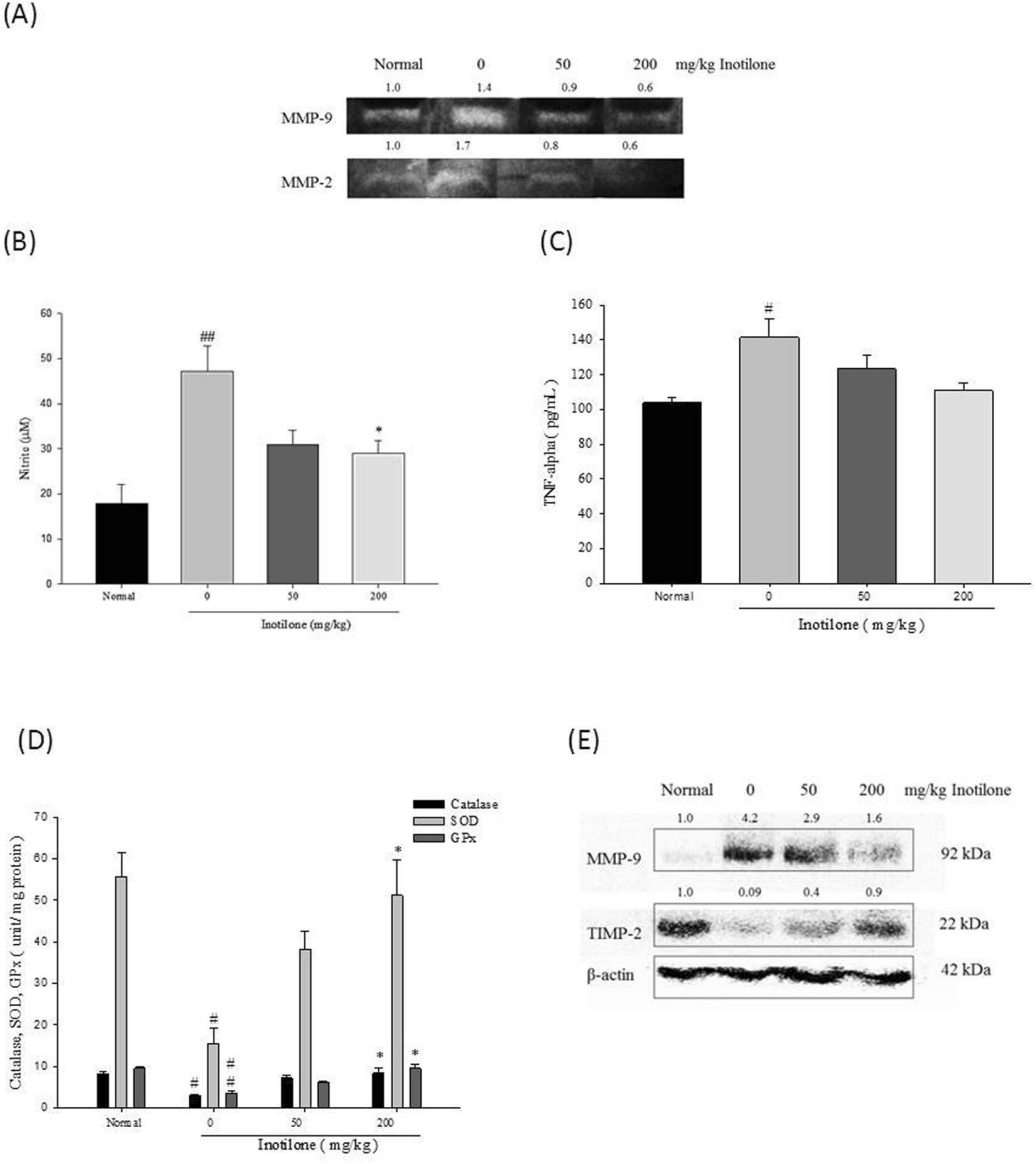
Effects of inotilone revealed the anti-cancer metastasis efficacy. (**A**) Gelatin zymography analysis revealed the activity of MMP-9 and MMP-2 in LLC-bearing mice serum. **(B)** The NO content in the serum of LLC-bearing mice was detected by Griess reagent. **(C)** The TNF-α expression was evaluated by a quantitative sandwich enzyme immunoassay technique. **(D)** The lung tissues were homogenized and centrifuged, and the supernatant was collected. The activities of catalase, SOD, and GPx in the supernatant were detected with ELISA assay. **(E)** The protein expression of MMP-9 and TIMP-2 in the lung tissues of LLC-bearing mice. Data represent the mean ± SD from at least three replicates with different mice. Statistical significance was analyzed by one-way ANOVA (^#^*p* < 0.05, ^##^*p* < 0.01 compared with the normal group; *p < 0.05 compared with the LLC-induced group).

In the normal group, MMP-9 activity in the serum was not observed compared to MMP-2. The serum activities of both MMP-2 and -9 in the tumor-induced mice were at higher levels compared to the normal group. The inotilone treatment groups had decreased serum MMP-2 and -9 activity, and the most significant reduction was observed in the 200 mg/kg group (Fig. 3A). NO and TNF-α are both inflammatory mediators associated with cancer metastasis. The results showed that both NO (Fig. 3B) and TNF-α (Fig. 3C) were significantly increased in the tumor-induced group. In contrast, the NO and TNF-α concentrations were decreased in a dose-dependent manner in the inotilone treatment groups.

### Effects of inotilone on antioxidant enzymes in the lung tissues of LLC-bearing mice

Metastatic progression of cancer is associated with changes in antioxidant enzymes^16^. Superoxidase dismutase (SOD), catalase, and glutathione peroxidase (GPx) are all ROS-scavenging enzymes. The results showed that SOD, catalase, and GPx were all significantly decreased in the tumor-induced group compared to the normal group (Fig. 3D). In contrast, when treated with inotilone, the enzyme levels were effectively restored. The results of the cell and animal experiments are consistent with each other. We propose that inotilone attenuates cancer metastasis in part by restoring the expression of antioxidant enzymes.

### Effects of inotilone on MMP-9 and TIMP-2 protein expression in the lung tissues of LLC-bearing mice

According to the results (Fig. 3E), in the normal group, MMP-9 protein expression in lung tissue was very low. However, the cancer-induced group mice had a higher level of MMP-9 compared to the normal group. The opposite was observed for TIMP-2 protein expression, with fairly high levels of TIMP-2 protein expression in the normal group. However, in the cancer-induced group, TIMP-2 expression was significantly lower than that in the normal group. With the addition of inotilone supplementation, MMP-9 expression was decreased and TIMP-2 expression was greatly increased in the 200 mg/kg group. Interestingly, in lung tissues, we could not observe MMP-2 or TIMP-1 expression in all groups.

### Effects of inotilone on the activity of MMP-2 and MMP-9 *in vitro*

**Figure 4:**
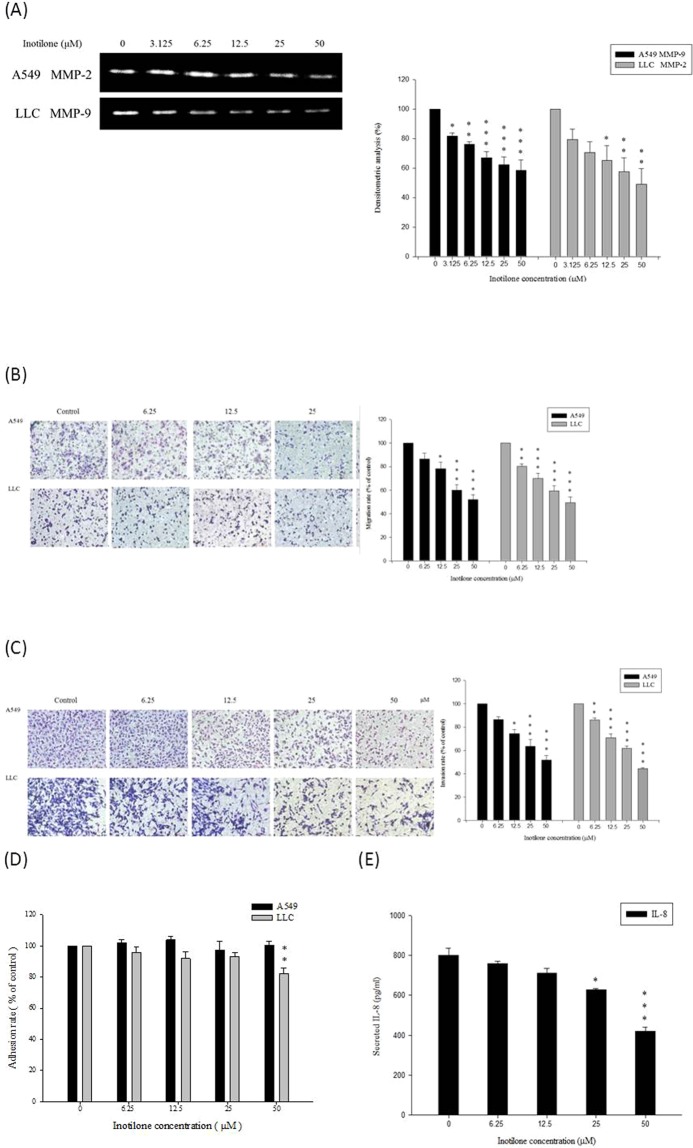
Inotilone suppressed cancer metastasis *in vitro*. (**A**) A549 and LLC cells treated with different concentration of inotilone for 24 h followed by collection of the supernatant for gelatin zymography analysis. The results revealed the activities of MMP-2 in A549 cells and MMP-9 in LLC cells. (**B**) In the migration assay, A549 and LLC cells were treated with various concentrations of inotilone (0, 6.25, 12.5, 25, and 50 μM) for 6 h. After migrating, the cells on the bottom side of the filter were fixed, stained and counted. All of the photographs were taken under 200x microscopic power fields. (**C**) The invasion assay in which the upper chambers were coating with Matrigel. A549 and LLC cells were treated with inotilone for 24 h at the concentrations given above, fixed, stained and counted as described above. (**D**) In the adhesion assay, the A549 and LLC cells were pre-treated with inotilone for 24 h at the above concentrations of inotilone. Then, the cells were seeded in 96-well plates coated with Matrigel and incubated for 2 h, and then, an MTT assay was used to determine the number of adherent cells. (**E**) The A549 cells were seeding in 96-well plates and treated with various concentrations of inotilone. The supernatant was taken to detect the IL-8 content using an ELISA kit. The data represent the mean ± SD from three independent experiments. *p < 0.05, **p < 0.01, and ***p < 0.001 compared to the control group.

The *in vivo* results revealed that inotilone had a great therapeutic effect in inhibiting cancer metastasis. To further explore the possible mechanism of inotilone suppression of cancer metastasis, we first investigated the efficacy of anti-cancer metastasis in A549 and LLC cells treated with inotilone. To examine the possible anti-metastatic mechanisms of inotilone, we determined the activities of MMP-2 in A549 and MMP-9 in LLC culture medium by gelatin zymographic analysis. Inotilone at 50 μM notably inhibited the activity of MMP-2 in A549 cells and the activity of MMP-9 in LLC cells (Fig. 4A). These results suggest that the anti-metastatic effect of inotilone is related to inhibition of the enzymatic degradative processes of cancer cell metastasis.

### Inhibitory effects of inotilone on cell migration, invasion and adhesion *in vitro*

The Transwell assay was used to evaluate the migratory and invasive ability of A549 and LLC cells after inotilone treatment. We found that inotilone added at 0–50 μM significantly decreased both the migratory (Fig. 4B) and invasive ability (Fig. 4C) of A549 and LLC cells. However, inotilone did not affect the adhesive ability of A549 cells under the same experimental conditions but had a slightly inhibitory effect of 17.7% on LLC cells (Fig. 4D).

### Inotilone inhibited the production of IL-8 in A549 cells

IL-8 expression is correlated with the metastasis of tumors and angiogenesis in numerous xenografts. The release of cytokines into the cell culture medium was measured by ELISA assay. Inotilone significantly decreased the level of IL-8 in the A549 cell culture medium (Fig. 4E).

### Effects of inotilone on the protein expression of MMP-2 and -9 and TIMP-1 and -2 *in vitro*

**Figure 5:**
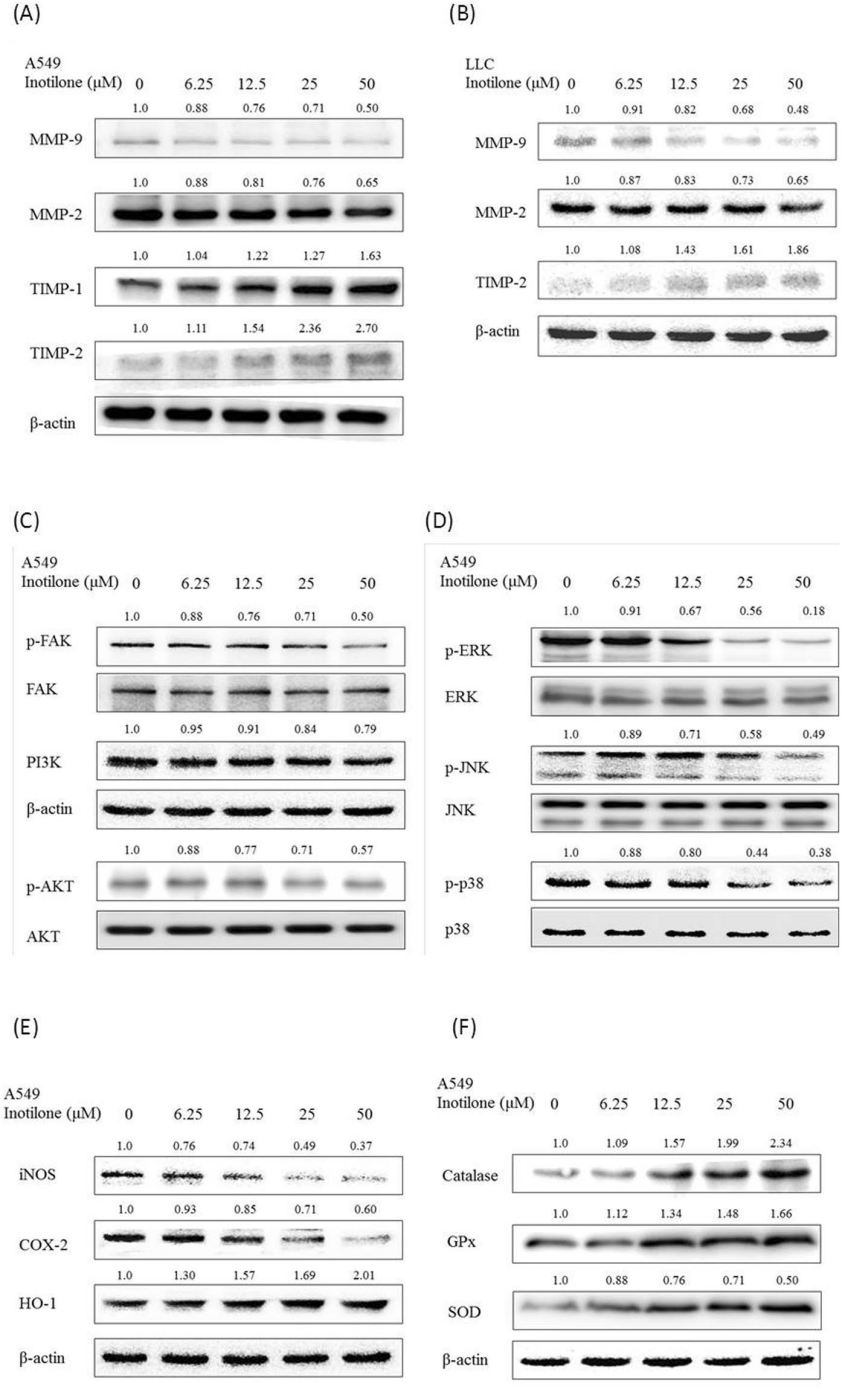
Inotilone affected protein expression as shown in western blots of A549 and LLC cells. A549 cells were treated with 0, 6.25, 12.5, 25, and 50 μM of inotilone for 24 h, and cell lysates were subjected to SDS-PAGE followed by western blotting and subsequently quantified by densitometric analysis. (**A**,**B**) Inotilone affected A549 and LLC cell protein expression levels of MMPs/TIMPs. (**C–E**) Inotilone inhibited the phosphorylation of AKT/FAK, MAPKs, and the inflammatory factors iNOS, COX-2, and HO-1. (**F**) Inotilone increased anti-oxidant enzyme protein expression such as for catalase, SOD and GPx. β-actin and each protein’s baseline expression level in the cell lysates were used as internal standards.

Inotilone inhibited MMP-2 and -9 expression with 35.2 and 49.6% decreases, respectively, at 50 μM in A549 cells. In addition, inotilone significantly increased TIMP-1 and -2 protein expression by 263.2 and 170.1%, respectively, at 50 μM (Fig. 5A). Similarly, inotilone also inhibited MMP-2 and -9 expression by 34.7 and 52.1%, respectively, at 50 μM in LLC cells and increased TIMP-2 protein expression by 86.1% at 50 μM (Fig. 5B). Based on these results, we propose that inotilone inhibition of cancer metastasis may be related to an imbalance between MMPs and TIMPs.

### The possible mechanisms of the inhibition of cancer metastasis by inotilone

There are many proteins that have been shown to be involved in cancer metastasis. First, several studies have reported that MAPK members are involved in the expression of MMPs and can induce metastasis. Our results showed that inotilone decreased the phosphorylation of ERK, JNK, and p38 protein expression but that it did not alter the level of protein expression (Fig. 5D).

Second, there is some evidence that FAK is involved in cell migration. Our results showed that inotilone significantly suppressed the phosphorylation of FAK in A549 cells but did not change the expression level of FAK. The PI3K/AKT pathway has also been identified as a major regulator of cellular proliferation, differentiation, and death in multiple cell types^17^. The results show that incubating A549 cells with inotilone led to dose-dependent decreases in PI3K levels and phosphorylation of AKT levels (Fig. 5C).

Third, the functional relationship between inflammation and cancer is not well known^11^. In many studies, overexpression of iNOS and COX-2 has been observed in many malignant tumors. In contrast, HO-1 is a protein that has a cytoprotective effect by reducing cell oxidative stress. We found that inotilone could decrease both iNOS and COX-2 protein expression in a dose-dependent manner. The opposite result was found for HO-1; inotilone increased HO-1 protein expression in a dose-dependent manner (Fig. 5E).

Fourth, there is evidence that cancer metastasis can be inhibited by targeting antioxidant enzymes. Our results showed that inotilone could significantly increase protein expression of catalase, SOD and GPx (Fig. 5F). These results confirm the connection between antioxidants and cancer cell metastasis.

**Figure 6:**
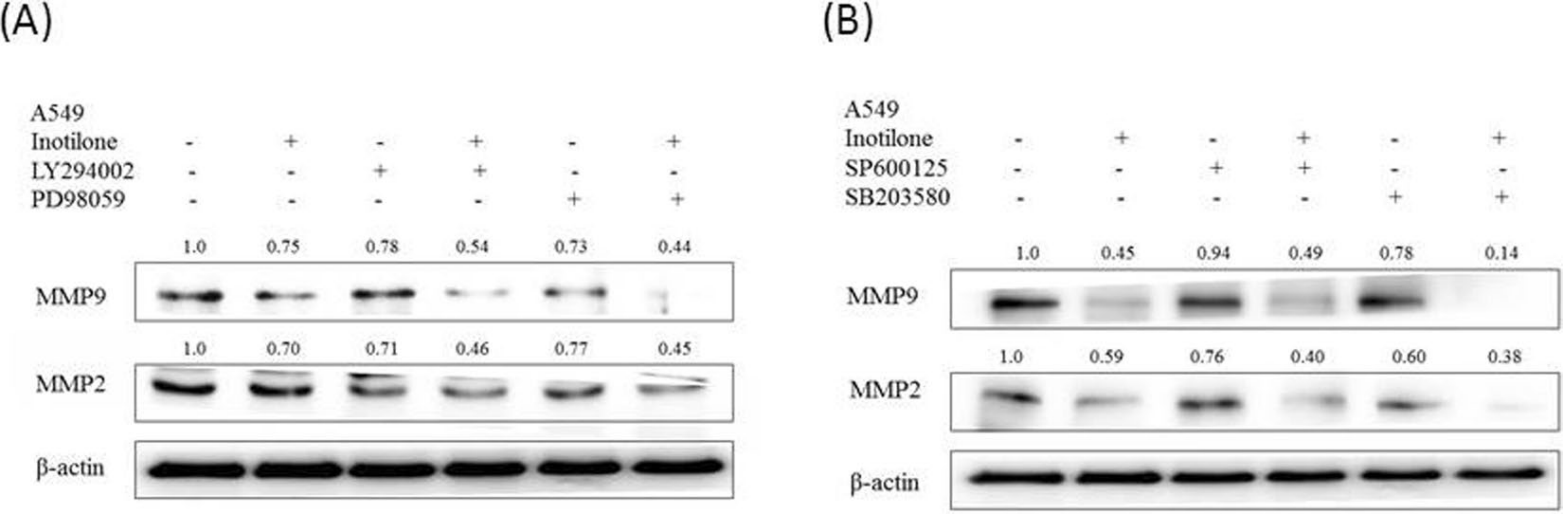
Inotilone inhibits IκBα phosphorylation and NFκB p65 nuclear translocation in A549 cells (**A,B**). A549 cells were treated with 0, 6.25, 12.5, 25, and 50 μM inotilone for 1 h. Total cells were harvested and fractionated into cytoplasmic and nuclear fractions by using a commercial product (Pierce Biotechnology, Rockford IL, USA, 50 reactions). The protein expression was evaluated with SDS-PAGE followed by western blotting and subsequently quantified by densitometric analysis. The protein expression levels of β-actin and α-tubulin were used as internal standards in each cytoplasmic and nuclear fraction.

Finally, NFκB, a transcription factor, is sequestered in the cytoplasm when binding to the IκB family and is activated by IκBα phosphorylation with subsequent degradation in proteasomes^18^. After treatment with inotilone, we observed that inotilone affected the phosphorylation of IκBα in the cytoplasmic fraction and induced nucleus translocation of NFκB in A549 cells (Fig. 6A,B).

### Inotilone inhibition of MMP-2 and MMP-9 using specific inhibitors to analyze the signaling transduction

**Figure 7:**
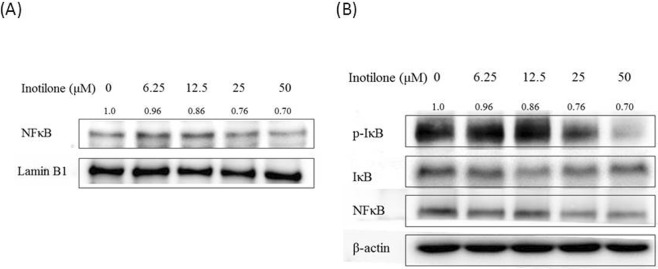
The protein expression of MMP-9 and MMP-2 was affected by MAPK inhibitors or co-treatment with inotilone in A549 cells (**A**,**B**). A549 cells were treated with PI3K inhibitor (LY294002), ERK inhibitor (PD98059), JNK inhibitor (SP600125) and p38 inhibitor (SB203580) alone or co-treated with inotilone 50 μM for 24 h, and cell lysates were subjected to SDS-PAGE followed by western blotting and subsequently quantified by densitometric analysis. The protein expression levels of β-actin and in cell lysates were used as internal standards.

To confirm whether the inhibition of cancer cell metastasis by inotilone occurs through PI3K, ERK, JNK and/or p38 suppression, A549 cells were pretreated for 1 h with PI3K inhibitor (LY294002; 50 μM), ERK inhibitor (PD98059; 50 μM), JNK inhibitor (SP60025; 50 μM) or p38 inhibitor (SB203580; 50 μM) and then examined for the effects of inotilone treatment. The results revealed that MMP-2 and -9 protein expression were decreased when given individual treatment with inotilone or each inhibitor. Furthermore, the combined treatment with each inhibitor and inotilone significantly reduced MMP-2 and -9 protein expression compared to inotilone or each inhibitor alone (Fig. 7A,B). In summary, the inhibition of MMP-2 and -9 by inotilone treatment is possibly caused by modulation of downstream signaling proteins, including PI3K, ERK, JNK, and p38.

## Discussion

Lung cancer is one of the most common and fatal forms of malignancy in Taiwan, with tumor cell metastasis being the primary cause of the poor prognosis. Extracts of *Inonotus linteus* (IL), a traditional medicinal mushroom, have been reported to have anti-proliferative and anti-metastatic effects^19–21^. In this study, we demonstrated that inotilone, which is one of the bioactive compounds found in extracts from IL, could reduce lung cancer metastasis both *in vitro* and *in vivo*.

Because there is little prior research on inotilone, we used different cell lines to investigate its cytotoxicity. Inotilone showed no significant cytotoxicity in cell lines other than A549 and LLC, such as Cal27 and HepG2 (Fig. S1). Next, we investigated the effect of inotilone on lung cancer metastasis *in vivo*. LLC cells in the C57BL6 mouse is a well-characterized model of metastasis^22^. There are two commonly used methods to induce cancer metastasis. One is direct injection of LLC cells into the bloodstream, also called experimental metastasis; the other is subcutaneous injection of LLC cells into the right or left flank of each mouse, also called spontaneous metastasis^23^. The experimental metastasis (intravenous injection) model is used for evaluating growth of malignant tumors in distant organs, but the spontaneous metastasis (subcutaneous injection) is the model used for observing malignant spread and tumor microenvironments^22,24^. Because our aim was to investigate anti-metastasis and observe malignant spread during treatment with inotilone, we chose the subcutaneous injection of LLC cells model.

The study of C57BL6 mice with subcutaneously injected LLC cells showed that inotilone could improve the survival rate of the mice and decrease the levels of proinflammatory cytokines TNF-α and NO in the serum for the highest dose inotilone group (Fig. 2A,B). Additionally, antioxidant enzymes in lung tissues were all increased in the high dose inotilone group (Fig. 3D). Moreover, histopathological data confirmed the anti-metastatic activity of inotilone in the lungs of LLC-inoculated mice (Fig. 2C,D). In addition, in the inotilone treatment group, MMP-2 and -9 enzyme activities were all decreased compared with the control group (Fig. 3A). The *in vivo* results demonstrated that inotilone had significant anti-metastasis efficacy, so we used cell experiments to explore the anti-metastatic mechanisms of inotilone.

Tumor metastasis and angiogenesis require controlled degradation of ECM, and an increase in MMP expression is associated with tumor invasion and metastasis of malignant tumors^9^. The activity of MMP-2 in A549 cells was inhibited at 3.125 μM, and the inhibition increased in a dose-dependent manner. A similar result was observed for MMP-9 in LLC cells (Fig. 4A). When A549 and LLC cells were treated with non-toxic doses, their migration and invasion were inhibited (Fig. 4B,C). These above results imply that the anti-metastatic effect of inotilone is associated with inhibition of enzymatically degradative processes.

Interleukin (IL)-8 is a proinflammatory CXC chemokine. IL-8 signaling potentiates the migratory ability of cancer cells, endothelial cells, and infiltration neutrophils at the tumor site. Recent studies conducted in ovarian and lung cancer cell lines showed that IL-8 signaling transactivates the epidermal growth factor receptor and activates downstream mitogen activated protein kinase (MAPK) signaling by mediating the growth factor receptor binding protein2/SOS-promoted activation of monomeric small G-protein, Ras-GTPase^25^. Furthermore, there are some studies that have shown that increased phosphorylation of Src-kinases and focal adhesion kinase (FAK) have also been detected in cancer cells after stimulation with IL-8^26,27^. These findings indicate that IL-8 expression is correlated with cancer cell migration and invasion.

In addition to IL-8 expression, it has been demonstrated that phosphorylation of FAK and PI3K/AKT protein expression participate in metastasis^28^. FAK is a cytoplasmic kinase that regulates ECM and various integrin-mediated mechanisms, including the MAPK/ERK signaling pathway. There is some evidence that FAK promotes fibronectin-mediated lung cancer metastasis through activation of Src, ERK, PI3K, and AKT^29^. The PI3K/AKT signaling pathway is involved in many cellular processes, including cell survival, cell adhesion, and metastasis. Our results showed that inotilone can inhibit the phosphorylation of FAK and AKT but does not affect their expression levels (Fig. 5C). The MAPK family includes ERK1/2, JNK and p38. MAPKs have been implicated in cell proliferation, apoptosis and metastasis. On the other hand, it has been reported that activation of MAPK signaling can increase the expression of MMPs^30^. In our results, the expression levels of ERK, JNK and p38 were all unchanged, while the phosphate states of these three proteins were all decreased in response to inotilone in a dose-dependent manner (Fig. 5D). The abovementioned results suggest that inotilone inhibits metastasis of A549 cells in part through suppression of the PI3K/AKT, ERK, JNK and p38 signaling pathways.

In recent years, some researchers have found that the tumor environment plays an important role in tumor growth, progression, and metastasis^31,32^. Increasing evidence has shown that ROS in the tumor microenvironment may cause cancer cell metastasis^33^, and possible mechanisms involve aberrant expression of integrins and MMPs and suppression of anoikis, as indicated by *in vitro* studies^34^. Physiologically generated ROS are normally reduced by anti-oxidizing agents, such as GPx, SOD, and catalases. Consistent with these findings, our results revealed that treatment with inotilone activated anti-oxidative enzymes, including SOD, GPx, and catalases. Through further *in vitro* experiments, we also observed that inotilone could reduce iNOS and COX-2 protein expression in A549 cells (Fig. 5E). Inflammatory cells also produce soluble mediators, such as metabolites of arachidonic acid, cytokines, and chemokines, which act by further recruiting inflammatory cells to the site of the damage and produce more reactive species^35^. According to the above results, we speculate that the anti-metastatic effect of inotilone might be related to antioxidant enzymes, anti-inflammation and amelioration of the tumor microenvironment.

NFκB is a heterodimeric protein consisting of p65 and p50 subunits, inactivated by binding to non-phosphorylated IκBα and activated when IκBα is phosphorylated. Activated NFκB translocates from the cytoplasm into the nucleus and regulates the expression of a wide variety of target genes, including MMPs^9^. Another study also described that NFκB is central in promoting cancer cell motility and invasion^36^. Our results demonstrate that inotilone can affect the levels of IκBα in the cytoplasm and decrease translocation of NFκB to the nucleus (Fig. 7A,B).

**Figure 8:**
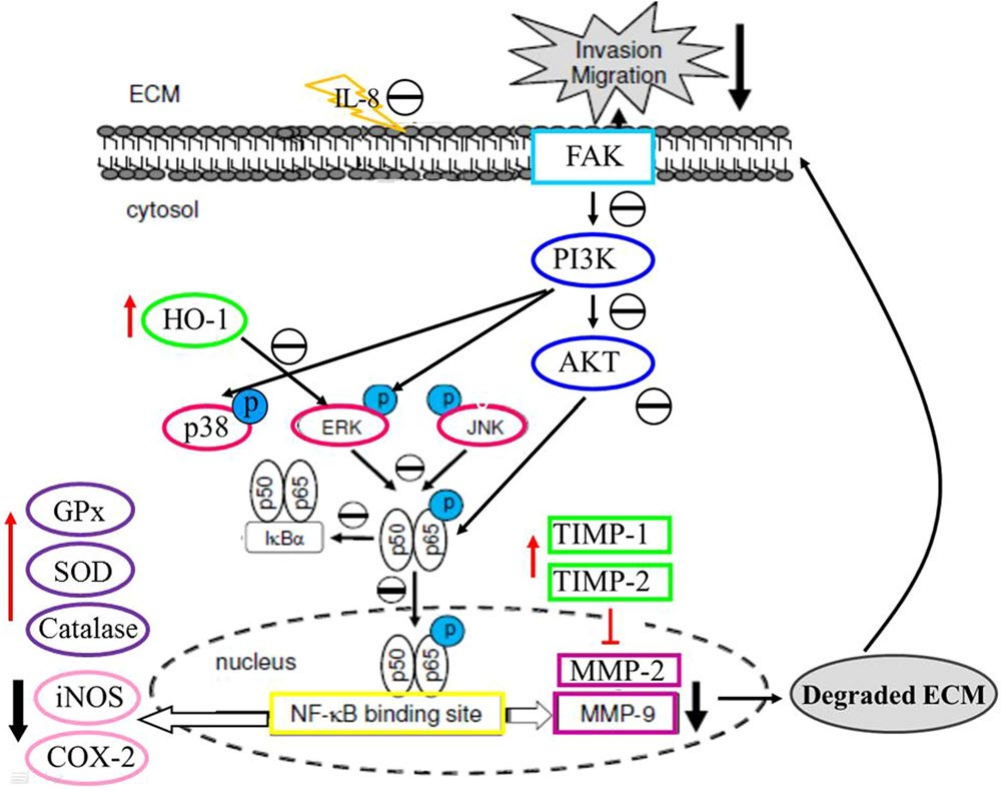
Anti-metastasis effect of inotilone and the proposed signaling pathway. The LLC-bearing mouse model revealed the anti-cancer metastasis efficacy of inotilone. Inotilone also ameliorated the inflammatory state of the tumor’s microenvironment. The signaling pathways of anti-cancer metastasis affected by treatment with inotilone in A549 cells. Inotilone significantly decreased MMP-2 and MMP-9 protein expression and activity, which may suppress IL-8 production and the FAK, PI3K/AKT, MAPK and NFκB pathways. In addition, inotilone enhanced anti-oxidative enzyme activation. The above results suggest that the anti-metastatic activity of inotilone may be mediated through regulation of the enzymatic antioxidant system and MMP protein expression.

In conclusion, the present study demonstrated that inotilone significantly inhibited the metastasis of transplanted LLC to the lungs of C57BL6 mice. The effects of inotilone are likely associated with decreased MMP-2 and MMP-9 protein expression and activity, which might suppress IL-8 expression and the FAK, PI3K/AKT, MAPKs and NFκB pathways. In addition, inotilone could also enhance the activity of anti-oxidative enzymes *in vivo* and *in vitro*, which suggested that some of its anti-metastatic activity might be mediated through regulation of the enzymatic antioxidant system (Fig. 8). According to the above, inotilone represents a potential therapeutic agent for the treatment of tumor metastasis from lung cancer.

## Methods

### Materials and chemical reagents

The human A549 lung adenocarcinoma and mouse Lewis lung carcinoma (LLC) cell lines were purchased from the Food Industry Research and Development Institute, Hsin Chu, Taiwan. F-12 Nutrient Mixture (Ham), 3-(4,5-dimethylthiazolyl-2)-2,5-diphenyltetrazolium bromide (MTT) and other chemicals were obtained from Sigma Chemical Co. (St. Louis, MO). Trypsin-EDTA, fetal bovine serum and penicillin/streptomycin were from Gibco Life Technologies, Inc. (Paisley, UK). Cell culture supplies were purchased from Costar (Corning, Inc., Cypress, CA). The human IL-8 ELISA kit was purchased from eBioscience (San Diego, USA).

### Isolation and determination of the active compound

The fruiting body of IL (approximately 1.5 kg, air dry weight) was powdered and extracted with 6 L 95% ethanol at room temperature (3 times, 72 h each). Extracts were filtered, combined together and then evaporated at 40 °C (N-11, Eyela, Japan) to dryness under reduced pressure to give a dark brown residue (60 g). The yield obtained for IL was approximately 4%. The crude extract was suspended in H_2_O (1 L) and then partitioned with 1 L *n*-hexane (x2), 1 L Ethyl Acetate (EA) (x2) and 1 L *n*-butanol (x2), successively. It yielded five fractions, an *n*-hexane soluble fraction, an EA soluble fraction, an *n*-butanol soluble fraction, a suspended fraction, and a water soluble fraction.

The active component was purified from the EA soluble portion. A portion of the active EA fraction was subjected to silica gel chromatography using stepwise CHCl_3_-MeOH (9:1, 8:2, 1:1 *v/v*) as the eluent. Final purification was achieved by preparative HPLC (Spherisorb ODS-2 RP18, 5 μm (Promochem), 250 × 25 mm, acetonitrile-H_2_O (83:17 *v/v*), at a flow rate of 10 mL/min and UV detection at 375 nm), and then, the fraction was recrystallized from EA to give inotilone.

### Cell culture

The human A549 lung adenocarcinoma and mouse Lewis lung carcinoma (LLC) cell lines were cultured in F-12 and DMEM, respectively, containing 10% (*v/v*) fetal bovine serum (FBS) and 100 U/ml penicillin/streptomycin, and incubated in a 5% CO_2_ humidified incubator at 37 °C. After cell confluence was reached, we used trypsin-EDTA to release the cells from the Petri dish and seeded the appropriate concentration of cells to maintain cellular proliferation.

### Animals and Cancer Metastasis Induction

Four-week-old C57BL/6 male mice were purchased from BioLasco Taiwan Co., Ltd. All animals and experiments were performed in accordance with the National Institutes of Health Guide for the Care and Use of Laboratory Animals and the regulations of China Medical University. The animal use protocol listed below has been reviewed and approved by the institutional animal care and use committee, and the protocol number is 101–71-N. The mice were housed individually in cages with controlled temperature (25 ± 2 °C) and humidity (65 ± 5%) with 12 h light/dark cycles. After a one week adaptation period, mice were implanted subcutaneously with 1 × 10^6^/100 μL LLC cells mixed with iced Matrigel at a ratio of 2:1. Three days after subcutaneous injection of LLC cells, the mice were randomly divided into four groups (*n* = 8 per group) as follows: group 1, normal; group 2, cancer induced (only implanted with LLC cells); and groups 3 and 4, tumor cells implanted and treated with either a high or low inotilone dose (200 and 50 mg/kg/day, sustained for 21 days). During the adaptation period and the 21 day experimental period, the mice consumed a standard rodent diet and water ad libitum. The growth of tumors was measured by caliper once a week. After the animals were sacrificed at the end of the 21 day period, blood samples were collected and centrifuged to obtain serum for experiments. At the end of the experiment, all lung lobes of each mouse were observed to visualize the tumor metastasis to the lung. The lung tissues were divided into two parts: one part was fixed in 10% (*v/v*) formalin, embedded in paraffin wax and then sliced into 3 μm sections. After H&E and IHC staining, pathological changes of the lung tissue slices were observed under a light microscope. The results were displayed through digital camera systems, incorporating a variety of charge-coupled device (CCD) detector configurations. Another part was homogenized and centrifuged, with collection of the supernatant to perform other experiments.

### Measurement of NO and TNF-α in LLC-bearing Mice Serum

NO determinations were carried out in 80 μL aliquots of samples diluted with PBS and then mixed with 80 μL Griess reagent^37^. The serum nitrite was quantified by using sodium nitrate as a standard curve. The serum levels of TNF-α were determined by using a quantitative sandwich enzyme immunoassay technique kit according to the manufacturer’s instructions.

### Measurement of Antioxidant Enzymes in LLC-bearing Mice Lung Tissues

Lung tissue homogenates were collected for the estimation of catalase^38^, superoxidase dismutase (SOD)^39^ and glutathione peroxidase (GPx)^40^ enzyme activities to detect the antioxidant activity of inotilone. The concentrations of the antioxidant enzymes were expressed as U/mg protein.

### Cell viability

Each cell line was seeded in a 96-well plate and treated with various concentrations of inotilone for 24 and 48 h. After incubation, the medium was replaced with fresh medium that contained 0.5 mg/ml MTT and incubated for 4 h at 37 °C. Formazan crystals were dissolved by the addition of an isopropanol/HCl solution and measured spectrophotometrically at 570 nm.

### Gelatin zymography assay

The activities of MMP-2 and -9 in both the medium and serum were assayed by gelatin zymography according to the protocol developed by Kleiner and Stetler-Stevenson with minor modifications^41^. Briefly, the culture medium was collected and electrophoresed in an 8% SDS-PAGE gel containing 0.1% gelatin. In animal studies, the serum samples of the mice were pooled because of the limited serum volume and were diluted 1:40 with PBS immediately before the assay. After electrophoresis, gels were washed at room temperature with 2.5% (*v/v*) Triton X-100 and subsequently transferred to reaction buffer for enzymatic reactions containing 1% NaN_3_, 10 mM CaCl_2_ and 40 mM Tris-HCl, pH 8.0, at 37 °C with shaking overnight. Finally, the gel was stained with 0.25% (*w/v*) Coomassie blue in 10% acetic acid (*v/v*) and 20% methanol (*v/v*) and destained in 10% acetic acid (*v/v*) and 40% methanol (*v/v*). The relative MMP-2 and -9 activities were quantified by Kodak Molecular Imaging software (version 4.0.5, Eastman Kodak Company, Rochester, NY) and represented by their relative intensities.

### Cell migration and invasion assay

A cell migration assay was performed using Transwell chambers according to the method reported by Repesh with some modifications^42^. Briefly, Transwell chambers (Millipore) with 6.5 mm polycarbonate filters with an 8 μm pore size were used. The cells were suspended in 200 μL serum free medium with or without various concentrations of inotilone and placed in the upper Transwell chambers. The lower chamber was loaded with 600 μL medium containing 10% FBS and various concentrations of inotilone. The cell invasion assay was similar to the cell migration assay, except that each filter was coated with 100 μL Matrigel diluted 1:10 in cold medium to form a thin continuous film on the top of the filter, which was then dried in an incubator for 30 minutes. After incubation, the cells on the upper surface of the filter were completely rubbed off using a cotton swab. The cells on the lower surface of the filter were fixed in methanol and stained with dilute Giemsa solution. For each replicate, the cells in 6 randomly selected fields were determined, and the counts were averaged.

### Cell adhesion assay

The A549 and LLC cells were pre-incubated with inotilone at various concentrations for 24 h at 37 °C. Each well of the 96-well plate was coated with Matrigel diluted 1:10 in cold medium to form a thin continuous film and dried in an incubator. Then, the cells were adjusted to 2 × 10^4^ cells/100 μL in medium and incubated at 37 °C for 2 h. After incubation, the cells were removed, washed twice in PBS and incubated with MTT. The attached cells formed formazan crystals that were dissolved in formazan by the addition of isopropanol/HCl solution and measured spectrophotometrically at 570 nm.

### Enzyme-linked immunosorbent assay (ELISA)

A549 cells were cultured in 6-well plates and treated with various concentrations of inotilone. The IL-8 content in the culture medium was measured by ELISA using an anti-human IL-8 antibody and a biotinylated secondary antibody according to the manufacturer’s instruction. The results were measured at 450 nm with an ELISA reader.

### Western blotting analysis

In cell culture experiments, the cells were seeded in Petri dishes and treated with various concentrations of inotilone. Nuclear and cytosolic protein extracts were prepared according to the manufacturer’s protocol (Pierce Biotechnology, 50 reactions). The cell pellets were collected and lysed with ice-cold RIPA buffer (1% NP-40, 50 mM Tris-base, 0.1% SDS, 0.5% deoxycholic acid, 150 mM NaCl, pH 7.5). After incubation overnight at −20 °C, the samples were centrifuged at 12,000 × g for 15 min. For the *in vivo* test, lung tissues were homogenized in the protein extraction solution with protease inhibitors and centrifuged at 10000 *x* g for 10 min. The supernatants were frozen at −20 °C until use. Proteins (50 μg) from the supernatant were resolved on 10% SDS-PAGE and transferred onto nitrocellulose membranes. Nonspecific binding of the membranes was blocked with TBST containing 10% nonfat milk for more than 1 h. The membranes were incubated with appropriate dilutions of the specific primary antibodies followed by the appropriate horseradish peroxidase-conjugated, goat anti-mouse, or anti-rabbit IgG. The bands were visualized using an ECL chemiluminescent detection kit (Thermo). The band intensity on the scanned films was quantified using Kodak Molecular (Version 4.0.5, Eastman *Kodak* Company, Rochester, NY) imaging software and expressed as relative intensity compared to the control group.

### Statistical analysis

Values were expressed as the mean ± S.D. and analyzed using one-way ANOVA followed by Scheffe’s for comparisons of group means. All statistical analyses were performed using SPSS: a *P* value < 0.05 was considered statistically significant.

## Supplementary information

Supplementary information
