# Supplementary material for: Inotilone from Inonotus linteus suppresses lung cancer metastasis in vitro and in vivo through ROS-mediated PI3K/AKT/MAPK signaling pathways
Source: Sci Rep. 2019 Feb 20;9:2344. doi: 10.1038/s41598-019-38959-z (PMC6382761; doi:10.1038/s41598-019-38959-z)

**Inotilone from** [***Inonotus linteus***](http://www.google.com.tw/search?nord=1&biw=1024&bih=643&noj=1&site=webhp&q=phellinus+linteus&spell=1&sa=X&ei=WWxXU8GwDIuGkgXd9IGgCA&ved=0CCkQvwUoAA) **suppresses lung cancer metastasis *in vitro* and *in vivo* through ROS-mediated PI3K/AKT/MAPK signaling pathways**

**Wei Chao ^1,2^, Jeng-Shyan Deng ^3^, Pei-Ying Li ^4^, Yueh-Hsiung Kuo ^1^, and Guan-Jhong Huang ^1,^***

*^1^ School of Chinese Pharmaceutical Sciences and Chinese Medicine Resources, College of Chinese Medicine, China Medical University, Taichung 404, Taiwan*

*^2^ Graduate Institute of Medical Sciences, College of Medicine, Taipei Medical University, Taipei, Taiwan*

*^3.^Department of Health and Nutrition Biotechnology, College of Medical and Health Science, Asia University, Taichung 413*, Taiwan

*^4^School of Pharmacy, College of Pharmacy, China Medical University, Taichung 404, Taiwan*

*corresponding: gjhuang@mail.cmu.edu.tw

**Supplementary information**

**Supplementary materials**

**Antibodies used for western blotting**

Primary antibodies:

β-actin (1:5000), IκB (1:2000), JNK (1:2000), p–p38 (1:1000), FAK (1:500), PI3K (1:3000), p–AKT (1:2000), and MMP–9 (1:1000) are from Merck Millipore，USA.

LaminB1 (1:500), MMP–2 (1:500), COX–2 (1:500), AKT (1:1000), Catalase(1:500). GPX1 (1:1000) are from GenTex，USA.

NFκB (1:1000), p–JNK (1:1000), p38 (1:1000), iNOS (1:200), HO–1 (1:2000) are from Abcam，Cambridge.

p-IκB (1:1000),TIMP–2 (1:1000), ERK (1:1000) are from Cell Signaling，USA.

TIMP–1 (1:500) is from ThermoFisher，USA.

p–ERK (1:1000) and p–FAK (1:1000) are from Invitrogen，USA.

SOD (1:1000) is from BioVision.

These primary antibodies were dilute in 0.1％ TBST and stored in -20℃.

Secondary antibody:

Peroxidase–Labeled Affinity Purified Antibody To Mouse IgG and Rabbit IgG (1:5000) were all from KPL，USA.

**Supplementary results**

**Figure 1S. Cell viability of Cal27 cells, HepG2 cells, CL1-0 cells, HT29 cells and MDA-MB-231 cells treated with inotilone by using a typical MTT assay.** The Cal27 (Human squamous cell carcinoma), HepG2 (Human hepatocellular Carcinoma), CL1-0 (Human lung adenocarcinoma) and HT29 (Human colorectal adenocarcinoma) were seeded into 96–well plates and treated with different concentrations (0, 3.125, 6.25, 12.5, 25, and 50 μM) of inotilone for 24h, and cell viability was determined by MTT assay. Data represent mean ± SD from three independent experiments.


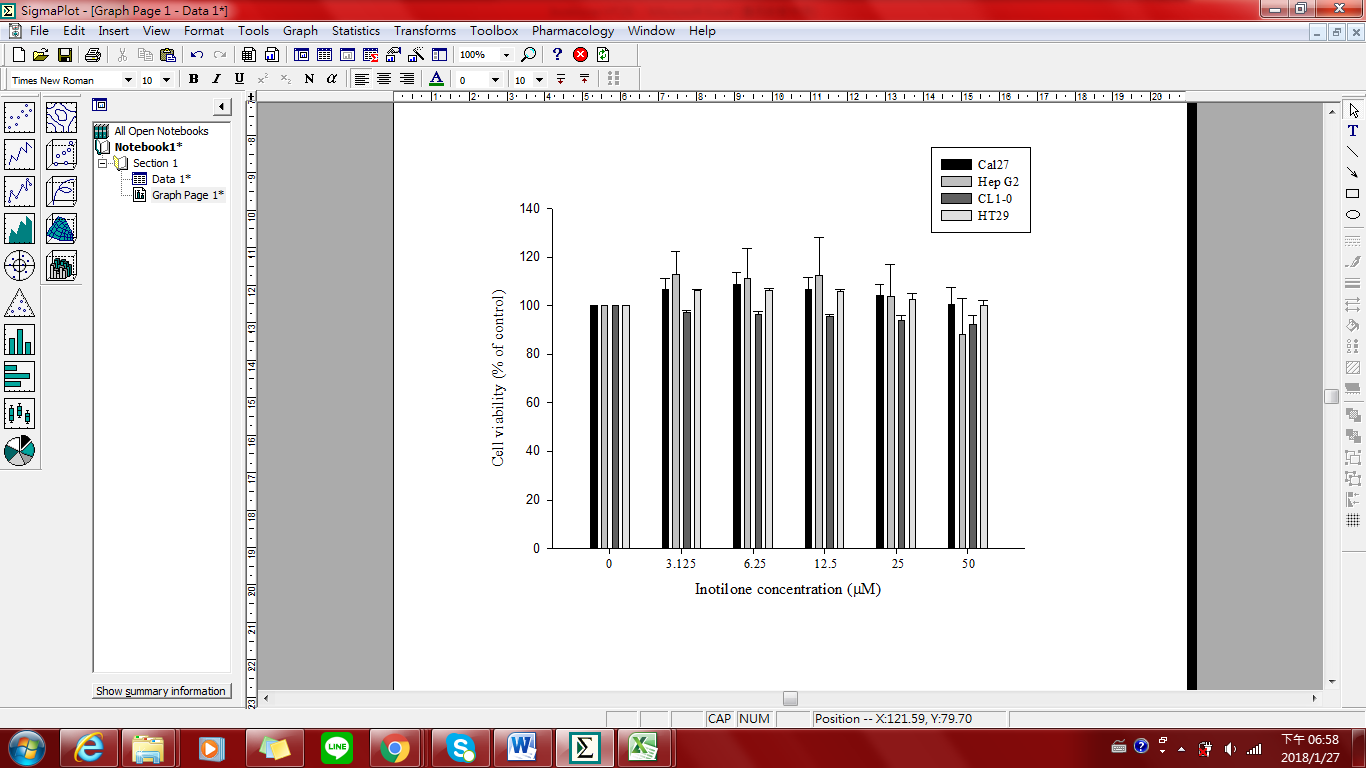


**Figure2S. The images of western blot analysis in A549 cells.** A549 cells were treated with 0, 6.25, 12.5, 25, and 50 μM for appropriate time. The cell lysates were added RIPA buffer or fractionated into cytoplasmic and nuclear fractions (A-S). The protein expressions of MMP–2 and MMP–9 were affected by treating PI3K inhibitor (LY294002), ERK inhibitor (PD98059), JNK inhibitor (SP600125), and p38 inhibitor (SB203580) or co–treating with inotilone (T-W).The proteins were used by SDS–PAGE and transfer into PVDF membrane. After conjugated with specific primary and appropriate second antibodies, the bands were visualized with chemiluminescent detection kit. The different protein expressions were performed in triplicate.

| (A)MMP-9 | (B)MMP-2 |
| --- | --- |
| **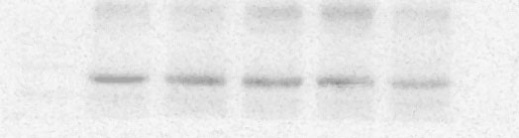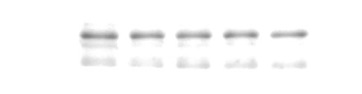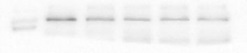** | **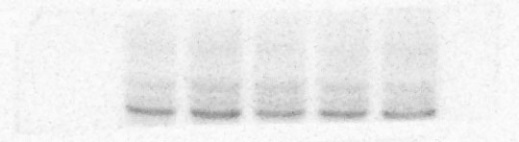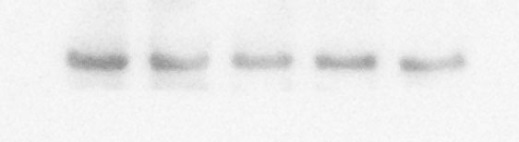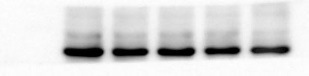** |
| (C)TIMP-1 | (D)TIMP-2 |
| **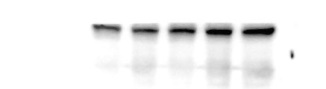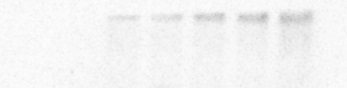**  **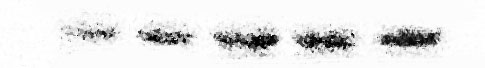** | **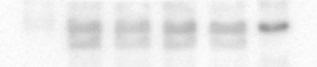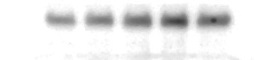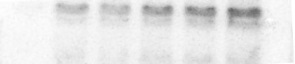** |
| (E)p-FAK | (F)PI3K |
| **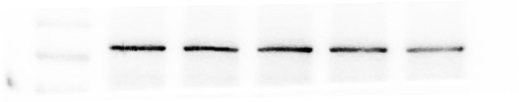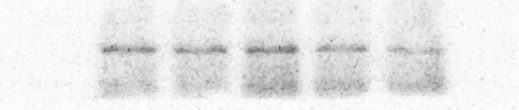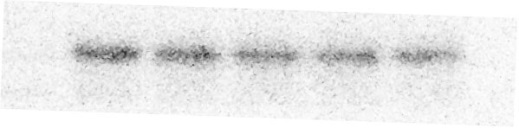** | **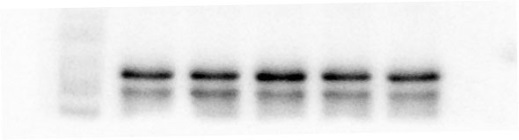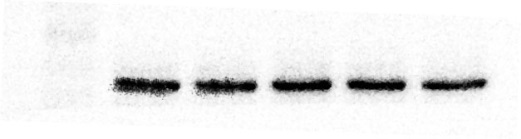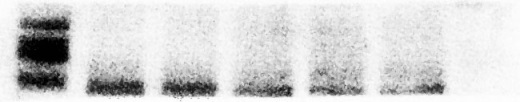** |
| (G)p-AKT | (H)p-ERK |
| **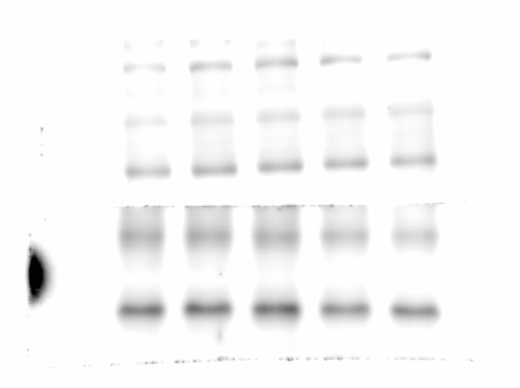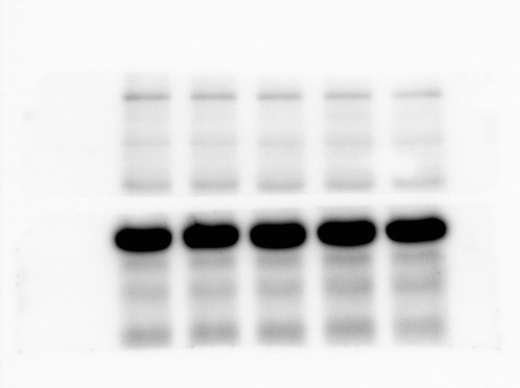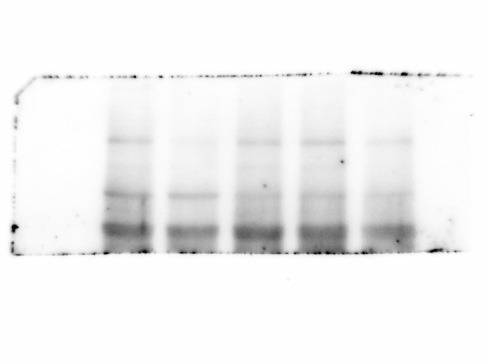** | **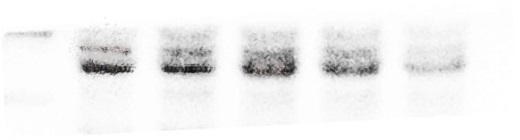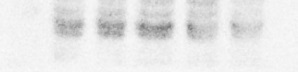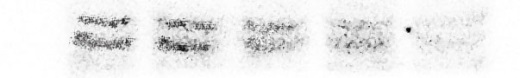** |
| (I)p-JNK | (J)p-p38 |
| **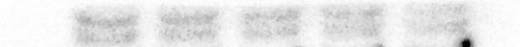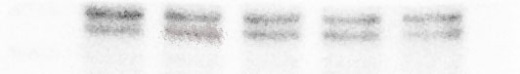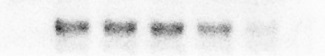** | **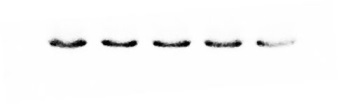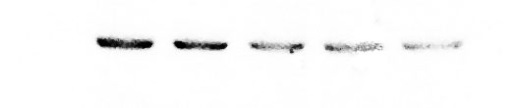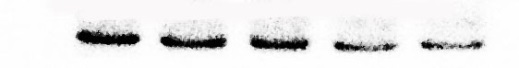** |
| (K)iNOS | (L)COX-2 |
| **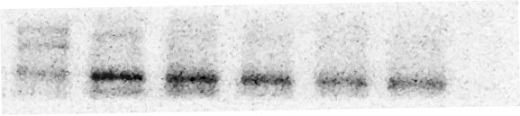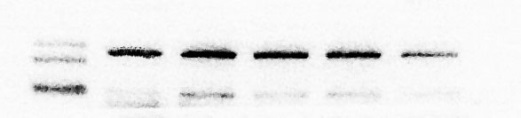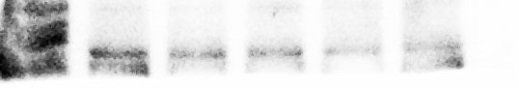** | **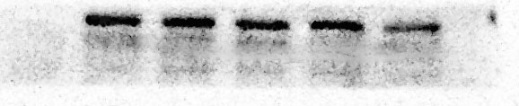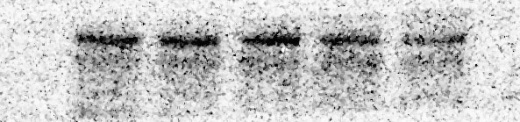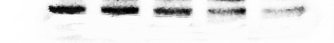** |
| (M)HO-1 | (N)Catalase |
| **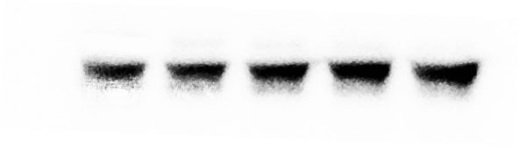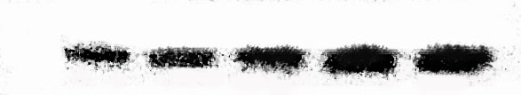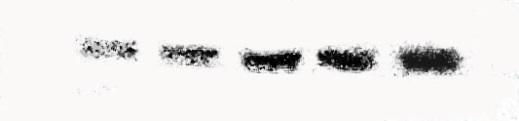** | **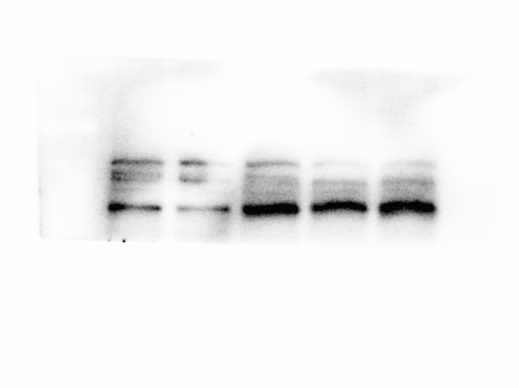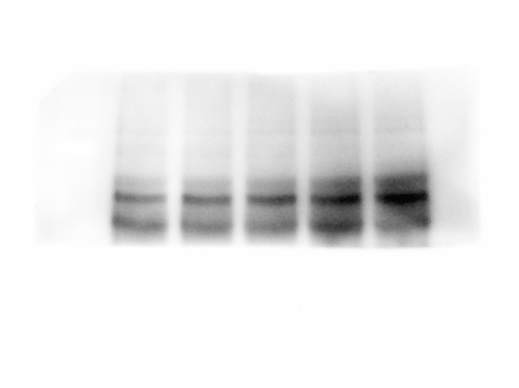** |
|  | 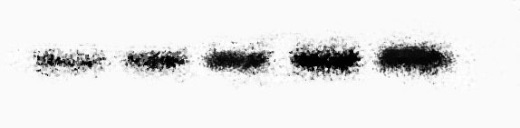 |
| (O)SOD | (P)GPx |
| **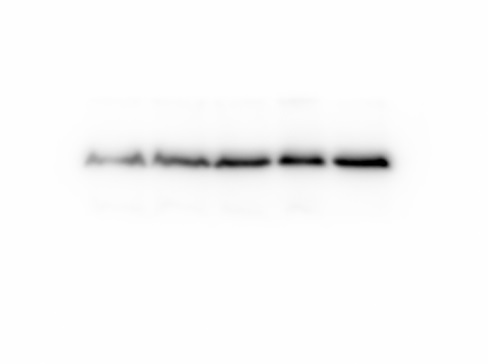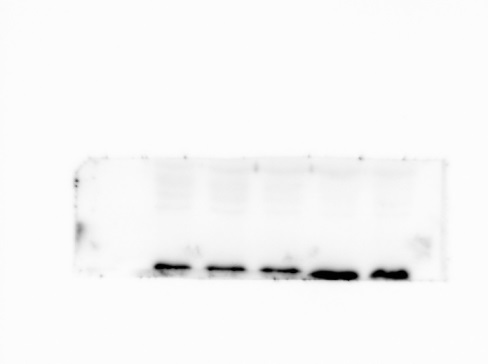** | **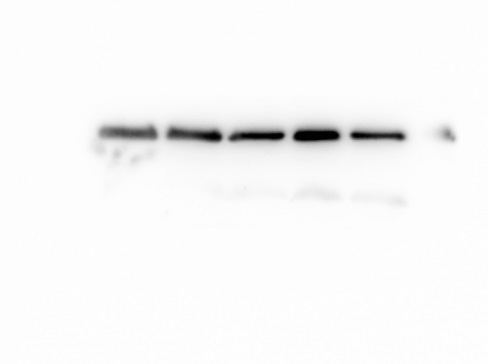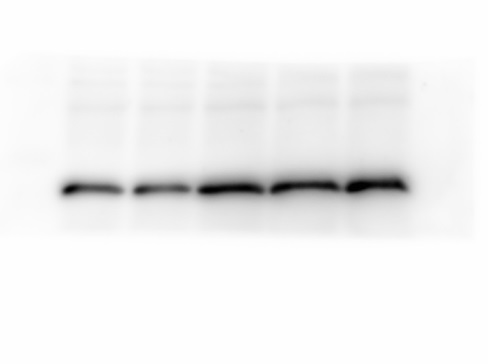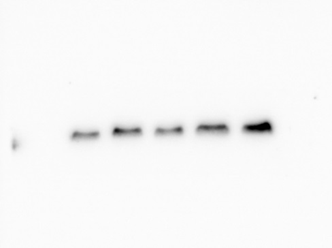** |
| (Q)nu-NFκB | (R)p-IκB |
| **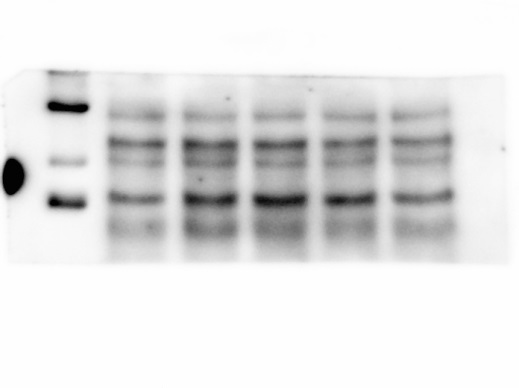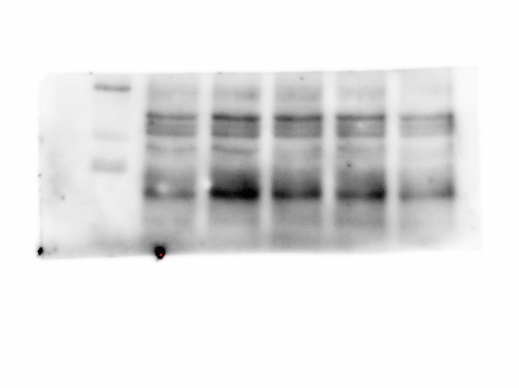**  **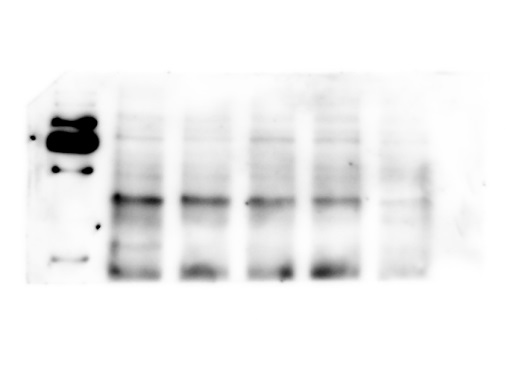** | 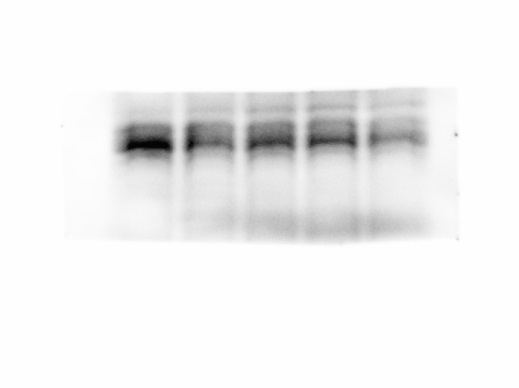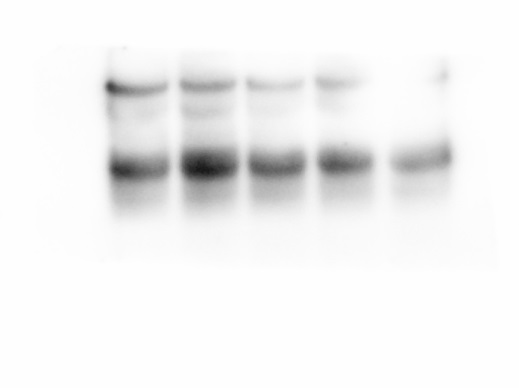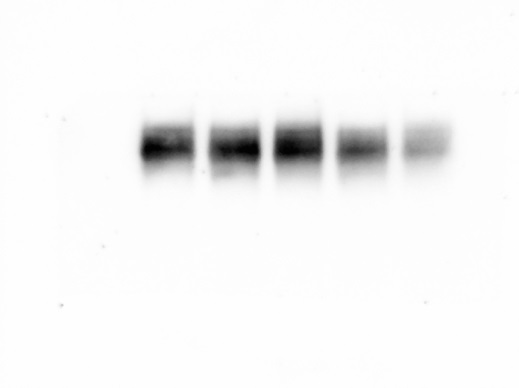 |
| (S)cyto-NFκB |  |
| 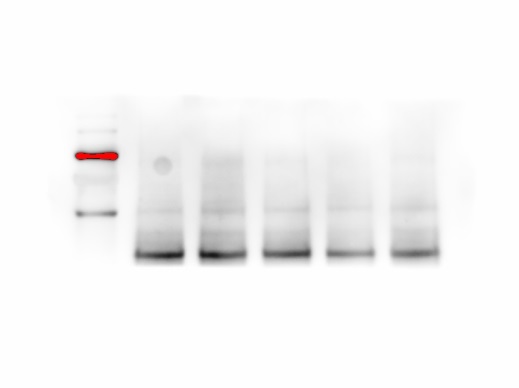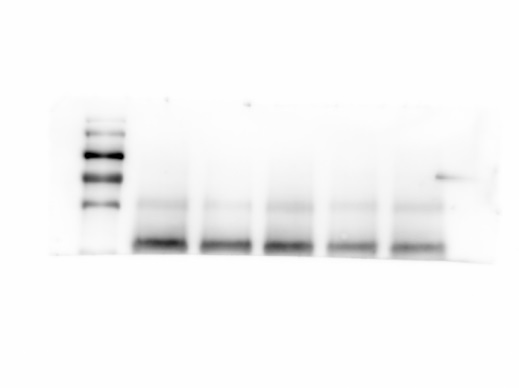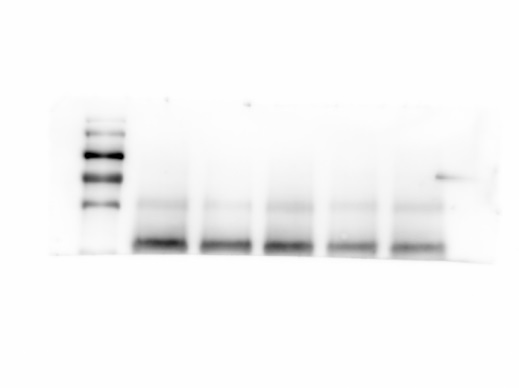 |  |
| (T)MMP-2-LY294002&PD98059 | (U) MMP-9-LY294002&PD98059 |
| **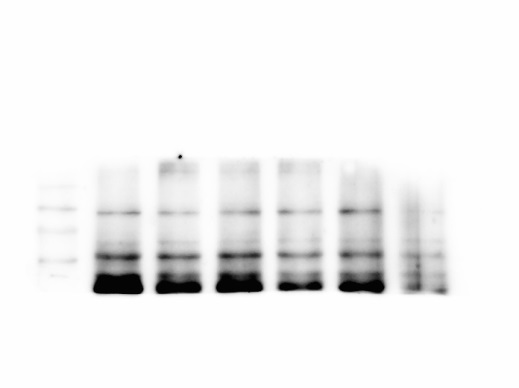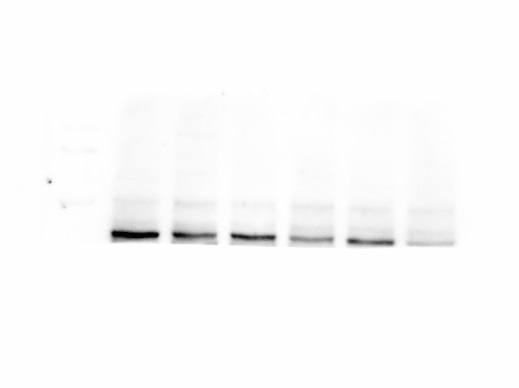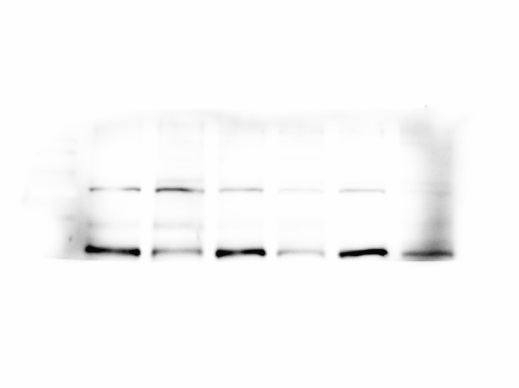** | **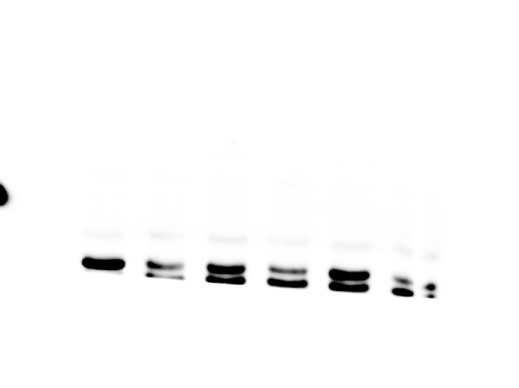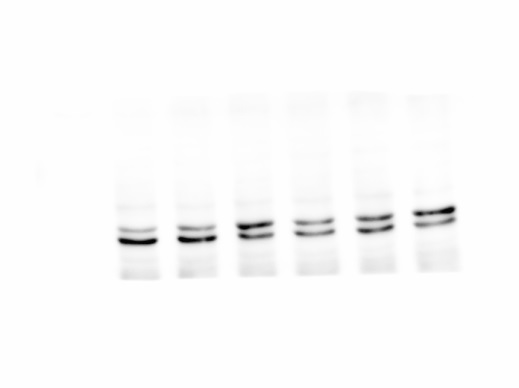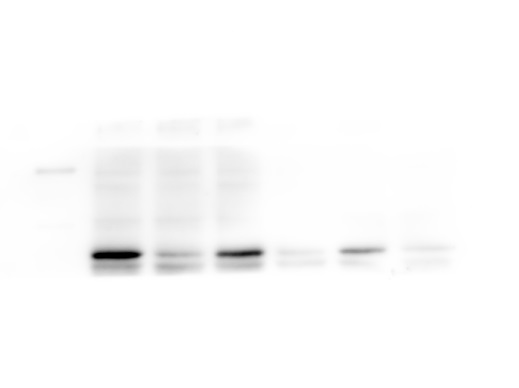** |
|  |  |
| (V) MMP-2-SP60025&SB203580 | (W) MMP-9-SP60025&SB203580 |
| **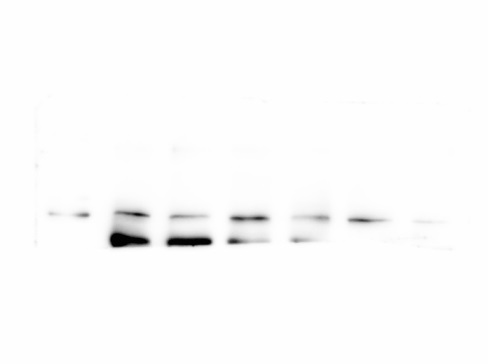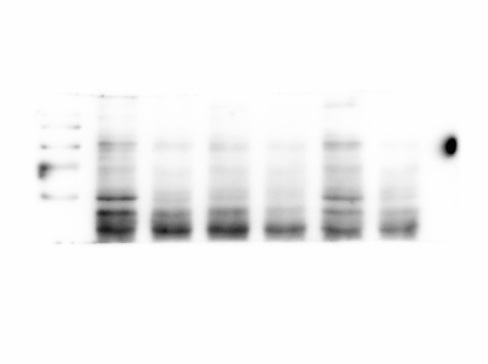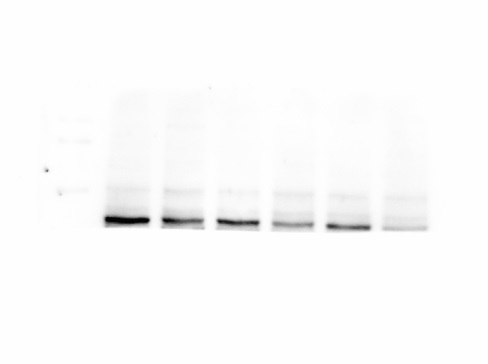** | **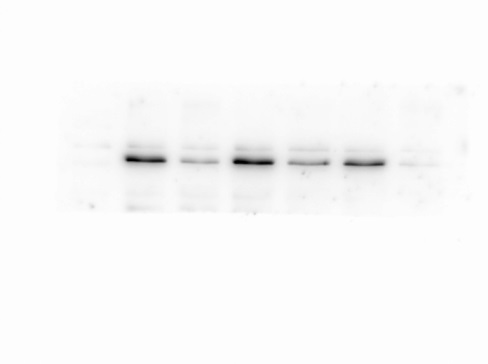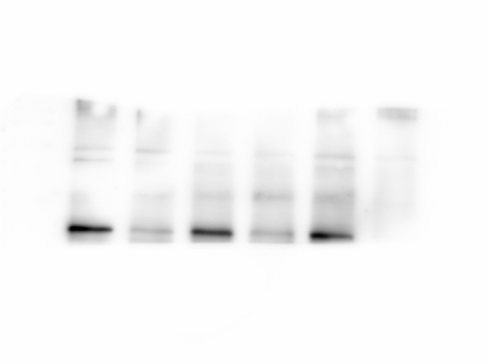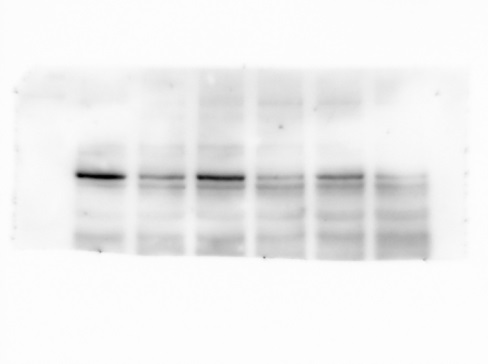** |

**Figure3S. The images of western blot analysis in LLC cells.** LLC cells were treated with 0, 6.25, 12.5, 25, and 50 μM for appropriate time. The cell lysates were added RIPA and quantities proteins were used by SDS–PAGE and transfer into PVDF membrane. After conjugated with specific primary and appropriate second antibodies, the bands were visualized with chemiluminescent detection kit. The different protein expressions were performed in triplicate.

| (A)MMP-9 | (B)MMP-2 |
| --- | --- |
| 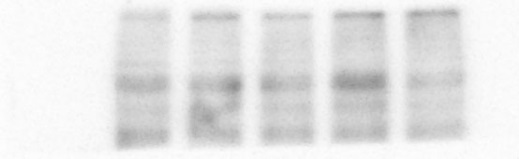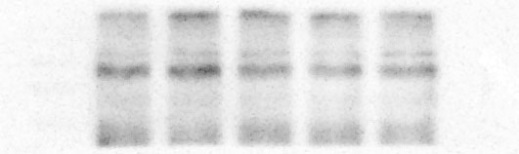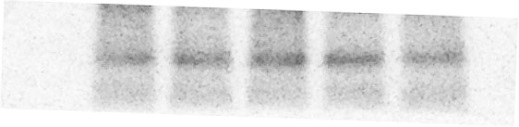 | 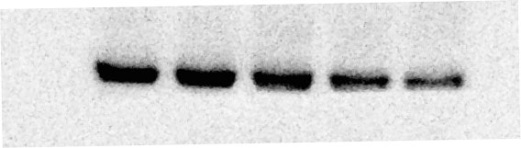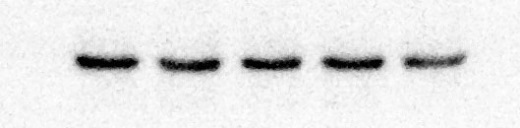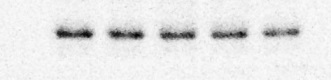 |
| (C)TIMP-2 |  |
| 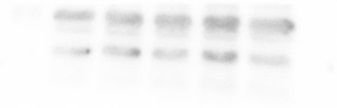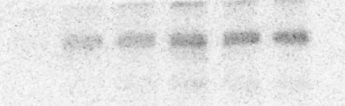 |  |


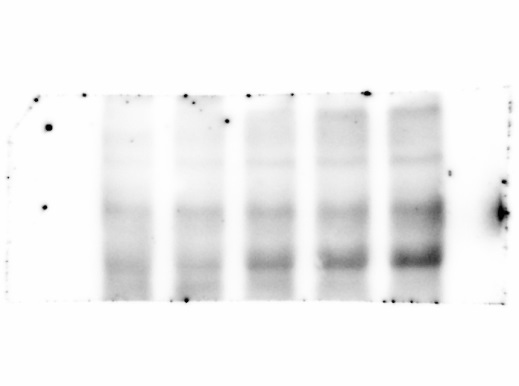

Supplement: Supplementary file 1 — Supplementary information [file 41598_2019_38959_MOESM1_ESM.docx]
